# The core microbiome of *Carya illinoinensis* (pecan) seedlings of different maternal pecan cultivars from the same orchard

**DOI:** 10.3389/frmbi.2022.1003112

**Published:** 2022-11-11

**Authors:** Kimberly Cervantes, Richard J. Heerema, Jennifer J. Randall

**Affiliations:** ^1^ Molecular Biology and Interdisciplinary Life Sciences, New Mexico State University, Las Cruces, NM, United States; ^2^ Extension Plant Sciences, New Mexico State University, Las Cruces, NM, United States; ^3^ Entomology, Plant Pathology, and Weed Science, New Mexico State University, Las Cruces, NM, United States

**Keywords:** microbiome, pecan, seedlings, pecan cultivars, leaves, stems, roots, seeds

## Abstract

*Carya illinoinensis* (pecan) produce a high valued and desired nut crop; with production expanding worldwide. Important attributes of pecans and their ability to adapt to different environments aside from their native regions are currently being investigated. Microbial communities are known to play an important role in crop productivity and overall plant health. Studies in other plant species have shown that the plant microbiome may be influenced by both the environmental conditions and genetics of the plant. Microbiota research in pecan is in its early stages and here we report insights into the core microbiome of seedlings derived from five pecan cultivars located in an orchard from Las Cruces, New Mexico. Seeds from open-pollinated pecan cultivars (‘Burkett’, ‘Mandan’, ‘Pawnee’, ‘Western’, and ‘Wichita’) were collected and grown in a quarantine greenhouse under highly regulated conditions. DNA from the resulting seedlings were used for next generation sequencing (MiSeq) for 16S and ITS and microbiome analyses revealed significant differences in microbial composition and relative abundance (bacterial and fungal) between seedling organs, with roots having the highest alpha diversity followed by stems and leaves. Bacterial family *Chitinophagaceae* was identified to be most relatively abundant in the roots of seedlings compared to the bacterial families *Rhizobiaceae* and *Moraxellaceae*, which were found to be most relatively abundant in the stems and leaves, respectively. Analyses also indicated that there were several families (bacterial families: *Rhizobiaceae*, *Enterobacteriaceae*, *Chitinophagaceae*, *Burkholderiaceae*, *Sphingomonadaceae*, *Pseudomonadaceae*, *Moraxellaceae*, *Microscillaceae*, *Rubritaleaceae*, *Caulobacteraceae*; fungal families: *Serendipitaceae*, *Nectriaceae*, *Ophiostomataceae*, *Hypocreaceae*, *Aspergillaceae*, and *Cephalothecaceae*) that were found in all seedlings and these constitute a core microbiome for pecan. There were also differences in microbial composition (bacterial and fungal) between seedlings from different maternal pecan cultivars and these differences are proposed to constitute a signature microbiome for the maternal cultivar. As pecan trees continues to extend to other growing regions it is important to understand the role that these microbes play in pecan. By establishing the core microbiome of pecans, the selection of microbes for breeding and improving pecan production will become a possibility.

## Introduction

*Carya illinoinensis* (pecan) possess genetic adaptations to grow in a broad environmental spectrum as its native region extends from Illinois, USA to Oaxaca, Mexico, making pecans a highly valued native nut crop within the United States with pecan production expanding worldwide. Commercial pecan (*Carya illinoinensis*) production in the United States began in the late 1800’s and, since then, efforts to improve pecan production have increased. There were several thousand different pecan genotypes with different phenotypes that spanned the native range. Improved cultivars have been developed through breeding efforts. Pecan cultivars in commercial orchards are currently grown and grafted onto rootstock to retain the desired plant parent characteristics. In addition, pecan tree cultivars differ in their ability to thrive under certain environmental conditions. Pecan cultivars may either be more susceptible or resistant to biotic and abiotic factors with geographical location influencing such susceptibility (Thompson and Young, 1985; Sparks, 1992; Lovell et al., 2021) (**Supplementary Table S1**). The genetic influences of each cultivar make them suitable for planting in specific geographical areas, and the role of their associated microbiome is yet to be understood. Diverse microorganisms, including bacteria, fungi, protozoa and viruses, are known to live in association with plants, either on the episphere or within the endosphere and collectively, these microbes form the microbiome. The source of a plant’s microbial composition arises from factors such as environment (including climate, insects, and soil), genetics (plant species, genotype, maternal plant and pollen (seed)), and management (Zalesny et al., 2005; Wang et al., 2012; Turner et al., 2013; Wagner et al., 2016; Manirajan et al., 2018; Bonito et al., 2019; Liu et al., 2019; Brown et al., 2020; Vishwakarma et al., 2020; Cordovez et al., 2021). Soil pH, soil organic matter, and soil temperature have also been correlated to microbial diversity and or the community structure in plant roots (Habiyaremye et al., 2020). The intensity of land use in agricultural practices, also influences bacterial community patterns in plants (Estendorfer et al., 2017). Additionally, seasonal patterns such as weather events and plant growth were found to alter maize root microbiomes (Walters et al., 2018).

The genetics of a plant not only defines its phenotype but also influences its microbiota as well. Specific studies within maize (*Zea mays*) have indicated that there are small but significant proportions of heritable variation of bacterial diversity in the rhizospheres of inbred maize (Peiffer et al., 2013; Walters et al., 2018). Additionally, specific operational taxonomic units (OTUs) were identified in three different potato varieties and distinct rhizosphere communities were also reported for different genetic clones of wild type and transgenic lines of *Populus* (Hacquard and Schadt, 2015; Fitzpatrick et al., 2018). Studies have also established that plants also acquire microorganism through vertical transfer from the maternal plant by means of vegetative tissues to the ovule, and into the developing embryo (Liu et al., 2017). Fort et al. (2021) identified the transmission of diverse fungal communities from maternal oak trees to the internal tissues of acorns (including the embryo), indicating the influence that maternal plants have in shaping the seed microbiome. Moreover, research by Moroenyane et al. (2021) found that the microbiome of soybean is modulated by the microbial communities present in the seed. Thus, the microbial communities identified in seedlings originate from a variety of sources including genetics and vertical transmission from mother-to-seed-to-seedling. Furthermore, as seeds germinate and seedlings grow, microbial communities change (Sugiyama et al., 2014). Plant age and developmental stages have been reported to select distinct microbiomes in the bacterial rhizosphere communities of *Arabidopsis* (Chaparro et al., 2014).

The microbiome of a plant can play an important role in its ability to uptake mineral nutrients, reduce its incidence of disease, as well as increase agricultural production (Richardson et al., 2009; Berendsen et al., 2012; Singh et al., 2020), thus, there is a great deal of interest among researchers on the soil and root microbiomes especially in agro-ecosystems. The root system may serve as a microbial reservoir, a bridge, in which microbes can be transferred horizontally from the microbe rich soil (Fierer, 2017; Vishwakarma et al., 2020). Arbuscular mycorrhizal fungi and rhizobium have been shown to facilitate nitrogen uptake in plants (Meng et al., 2015). A plant’s likelihood to thrive or fail under any condition is highly influenced by the microbes that inhabit the plant. The predominant bacteria and fungi in plant tissues of many species has been studied (Berendsen et al., 2012; Bulgarelli et al., 2012) and research has revealed a difference in abundance and presence/absence of microbial organisms among different plant tissues. Research on *Populus* by Cregger et al. (2018), indicated a difference of archaeal/bacterial and fungal communities across leaves, stems, roots, and soils. In addition, archaeal/bacterial diversity was found to be highest in the roots and soils followed by stems and leaves compared to fungal diversity, which was found to be highest in the stems and soils. Furthermore, recent studies exploring the bacterial diversity on *Paris polyphylla*, a traditional Chinese herb, also found bacterial communities to be significantly higher in the roots than those in the stems and leaves (Liu et al. (2020). Interestingly, no differences were found in leaf and root microbial richness and diversity of *A. thaliana* from four different field sites (Bodenhausen et al., 2013).

Plants are now recognized to have a specific heritable core microbiome. The core microbiome is defined as those species of microorganisms that remain constant within the microbiomes of all members (i.e., genotypes) of a given plant species despite plant growth stage, geographical location, genotype, etc. (Bhagat et al., 2021). The establishment of a heritable core microbiome is caused at least in part by the influence that the plant’s genetics has on the recruitment of microorganisms. The *Arabidopsis* core microbiome was reported in 2012 by Lundberg et al. In addition, the core microbiome of some monocots and dicots have been established (i.e., rice, wheat, coffee, beans, and switch grass) however, the core microbiome of pecans remains unknown (Fitzpatrick et al., 2018; Eyre et al., 2019; Grady et al., 2019; Pérez-Jaramillo et al., 2019; Fulthorpe et al., 2020; Kuźniar et al., 2020). In this study, seedlings from pecan nuts obtained from five maternal pecan cultivars were studied with the goal to identify and differentiate the microbial organisms present in pecan seedlings. Analyses in this study sought to determine what microbes were found in specific tissues and to begin to define the core microbiome of pecan. Doing so, will allow for future research on the roles and influences that microbes play in pecan processes.

## Materials and methods

### Seed and plant material collection

Fully developed seeds from five pecan cultivars (‘Burkett’, ‘Mandan’, ‘Pawnee’, ‘Western’, and ‘Wichita’) were collected November 2018 from the NMSU experimental farm Leyendecker. Within 8 days of collection, the seeds were planted in Lamberts professional high porosity growing mix (Québec, Canada) that was treated with hydrogen peroxide. Seedlings were grown in a quarantine greenhouse with a temperature of 29°-35°C at New Mexico State University, the Las Cruces campus for approximately three months with an average height of 22.2 cm (from the base of the plant). A total of 5 seedlings per cultivar were separated into leaf, stem, and root tissues; the soil on root tissues were thoroughly removed by washing. Samples were stored in a -20°C freezer for further processing.

### Genomic DNA extraction

The tissues from each seedling were homogenized using a mortar and pestle in liquid nitrogen, and transferred into microcentrifuge tubes. Samples were stored in a -20°C until further processing. The Qiagen DNeasy Plant Mini Kit (#69106, Qiagen, Hilden, Germany) was used to extract total gDNA according to the manufacturer’s protocol. The concentration and quality of the extracted DNA was measured using the NanoDrop 2000 ultraviolet (UV)-Vis spectrophotometer (ThermoFisher Scientific, Waltham, MA) and stored in TE buffer at -20°C to prevent DNA degradation.

### Next generation sequencing and microbiome analysis

DNA samples, with a concentration between 23.5 ng/μL – 100 ng/μL, were sent to the Genomics Center at the University of Minnesota (UMGC), Minneapolis, USA for next generation sequencing. The following Nextera compatible primers for the V4V6 16S rRNA and ITS2 region for bacterial and fungal analysis were used by UMGC, respectively: 16S V4 (TCGTCGGCAGCGTCAGATGTGTATAAGAGACAGGTGCCAGCMGCCGCGGTAA), 16S V6 (GTCTCGTGGGCTCGGAGATGTGTATAAGAGACAGCGACRRCCATGCANCACCT), ITS2 (TCGTCGGCAGCGTCAGATGTGTATAAGAGACAGTCGATGAAGAACGCAGCG), and (GTCTCGTGGGCTCGGAGATGTGTATAAGAGACAGTCCTCCGCTTATTGATATGC). Sequence pairing and trimming, quality control, OTU clustering, chimera filtering, alpha and beta diversity analysis, and statistical analysis of the sequencing results were performed using CLC Genomics Workbench 21.0.4 Microbial Genomics Module version 21.1. The parameters used followed the settings as configured by the workflow of the Microbial Genomics Module with some modifications. Paired-end reads had a minimum and maximum distance of 100 and 550, respectively, for both bacterial and fungal reads,. Illumina options included quality scores of NCBI/Sanger or Illumina Pipeline 1.8 and later, and the option to discard failed reads was selected. Read trimming included quality and adapter trimming, as well as sequence filtering. Bacterial and fungal reads were trimmed from the 3’-end to a fixed length of 210 and 220 bp, respectively. Samples were filtered based on a minimum number of reads of 100 with a minimum percent from the median of 25. The bacterial database SILVA 16S v132 along with the full UNITE v7.2 fungal database were used as references for operational taxonomic unit (OTU) clustering of sequencing reads. Clustering threshold was set to 97% similarity. Settings also included the creation of new OTUs with a taxonomy similarity percentage of 80, minimum occurrences of 1, fuzzy match duplicates, and find best match were also used. Furthermore, chimeric sequences were removed, and mitochondrial and chloroplast sequences were filtered for 16S analysis. For OTU clustering visualization of the fungal communities, unknowns were removed. The Microbial Genomics Module MUSCLE tool was used to construct a phylogenetic tree using a Maximum Likelihood approach with the Jukes Cantor nucleotide substitution model, based on a Multiple Sequence Alignment (MSA) of the OTU sequences for both bacterial and fungal communities. The resulting phylogenetic trees were used for alpha diversity analyses. A maximum sampling depth of 5,000 reads was used for rarefaction analysis for both bacterial and fungal communities. Vox plot visualization of alpha diversity was generated using CLC Microbial Genomics Module version 21.1. For beta diversity, Bray-Curtis was used as the dissimilarity metric. All bacterial and fungal sequence data were deposited at the NCBI Sequence Read Archive as part of BioProjects PRJNA796047 and PRJNA796064, respectively. The non-parametric Kruskal-Wallis and Mann-Whitney tests were used for alpha diversity comparisons. For beta diversity and principal coordinate (PCo) analysis, the Bray-Curtis dissimilarity metric was used along with the cmdscale function of the program language and software R. To quantify the influence and interaction of maternal cultivar and plant compartment on microbial communities, the Adonis test was performed using the adonis2 function in the vegan library of the software R. The Bray-Curtis dissimilarity metric was used as input for the adonis2 function which was computed using the vegdist function, also available in the vegan library. Two summary statistics to assess the importance of the sources of variation (in analysis of variance table) were computed in the adonis2 function; the R^2^ value (computed as the ratio of the Sum of Squares of the factors and the Total Sum of Squares), and p-value. Hierarchical clustering heat maps were generated using the 25 most different OTU’s across all samples (FDR p-value=0) with the CLC Microbial Genomics Module version 21.1. A permutational multivariate analysis of variance (PERMANOVA) analysis was conducted using the CLC Microbial Genomics Module version 21.1 to measure the effect size and significance of beta diversity between seedlings (including each plant compartment) within their respective maternal cultivar. To determine the core microbiome, OTUs aggregated at the taxonomic family level were analyzed for presence and abundance across maternal cultivars. Here, aggregated families shared across each maternal cultivar were determined as core microbiome. Inferred functions of OTUs respective to the core microbial families were conducted using PICRUSt2 and the CLC Microbial Genomics Module Infer Functional Profile tool. The EC functional term counts associated with 16S regions in prokaryotes and the ITS regions of fungi and the EC databases were used as parameters.

## Results

### Microbiome analysis of seedling tissue type

Sequence analysis of bacterial communities resulted in a total of 5,603 OTUs with the exclusion of mitochondria, chloroplast, unknowns, and ambiguous taxa. Of these, 658 were identified in the leaves, 846 in the stems, and 4,621 in the roots. A count of 110 OTUs were shared across all tissue types with 124 OTUs shared between leaves and stems; 166 shared between leaves and roots; and 342 shared between stems and roots. Of the 362 OTUs aggregated by family, 133 were identified in the leaves, 173 in the stems, and 340 in the roots. Analysis revealed 104 families were shared between leaves and stems; 127 families were shared between leaves and roots; and 157 families were shared between stems and roots. Of the 428,370 fungal OTUs analyzed, 17,974 were identified in the leaves, 239,193 in the stems, and 172, 273 in the roots with only 16 fungal OTUs shared across all tissue types. A count of 20 fungal OTUs were found to be shared between leaves and stems; 25 between the leaves and roots; and 41 between stems and roots. Of the 105 OTUs aggregated by family, 41 were identified in the leaves, 51 in the stems, and 91 in the roots. Analysis revealed 26 families shared between leaves and stems; 33 families shared between leaves and roots; and 44 families shared between stems and roots. Furthermore, fungal analysis revealed that of the 428,370 fungal OTUs, 402,803 were unknowns. For visualization purposes, unknowns were excluded from OTU tables (**Supplementary Figure S1B**).

Bacterial and fungal microbiome sequence analysis revealed differences in microbial population abundances and presence between pecan tissues (**Supplementary Figure S1**). Bacterial analysis revealed the family *Chitinophagaceae* (Bacteroidota) to be present in leaves, stems, and roots with roots having the most relative abundance. Conversely, the family *Rhizobiaceae* (Pseudomonadota) was also found in the leaves, stems, and roots with the stems having the highest relative abundance. *Moraxellaceae* (Pseudomonadota) was found to be most abundant in the leaves followed by the stems and roots with the least relative abundance. Other top bacterial families identified in pecan tissues include *Sphingomonadaceae* (Pseudomonadota)*, Pseudomonadaceae* (Pseudomonadota)*, Rubritaleaceae* (Verrucomicrobia)*, Microscillaceae* (Bacteroidota)*, Caulobacteraceae* (Pseudomonadota)*, Enterobacteriaceae* (Pseudomonadota), and *Burkholderiaceae* (Pseudomonadota). Fungal analysis identified the family *Serendipitaceae* (Basidiomycota) with the highest relative abundance in the roots followed by leaves and stems, respectively. The family *Nectriaceae* (Ascomycota) was found to be most abundant in pecan stems, with leaves and roots having similar relative abundance. Other top fungal families identified in pecan tissues include *Cephalothecaceae* (Ascomycota)*, Hypocreaceae* (Ascomycota)*, Aspergillaceae* (Ascomycota)*, and Ophiostomataceae* (Ascomycota).

### Alpha diversity analysis of seedling tissue type

**Figure 1:**
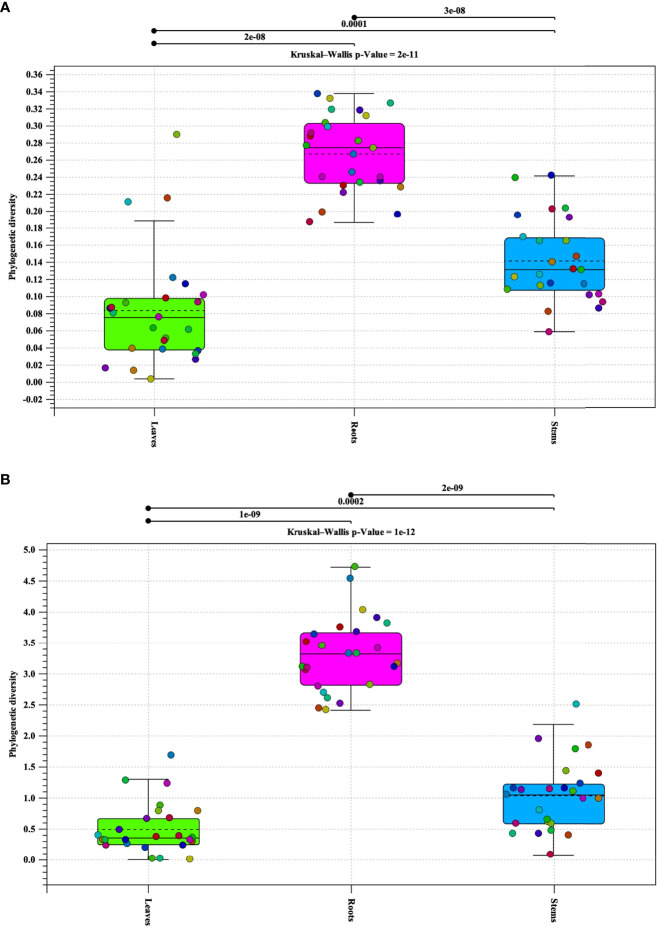
Phylogenetic alpha diversity of microbial populations in pecan tissue types. Box plot and graph visualization of bacterial **(A)** and fungal **(B)** alpha diversity in pecan. Colored dots indicate seedling samples per tissue type.

Alpha diversity analysis of bacterial populations revealed that roots have the highest diversity followed by stems and leaves, respectively (**Figure 1A**). The samples in each tissue type (leaves, stems, and roots), indicate a relationship of homogeneity among each other. Analysis indicated two outliers present in the leaf samples. Alpha diversity analysis of fungal populations indicated a similar pattern as that of bacterial populations (**Figure 1B**). Roots were found to contain the highest alpha diversity followed by stems and leaves. However, the alpha diversity identified in the roots, stems, and leaves samples were similar to each other. In addition, the samples in each tissue types continued to follow a relationship of homogeneity. Furthermore, fungal analysis of the leaves and stems revealed an outlier for each tissue type respectively. P-values for both bacterial and fungal analyses, indicate that there is sufficient evidence for difference in alpha diversity between tissue types (bacterial p-value = 2e-11 and fungal p-value = 1e-12).

### Beta diversity analysis of seedling tissue type

**Figure 2:**
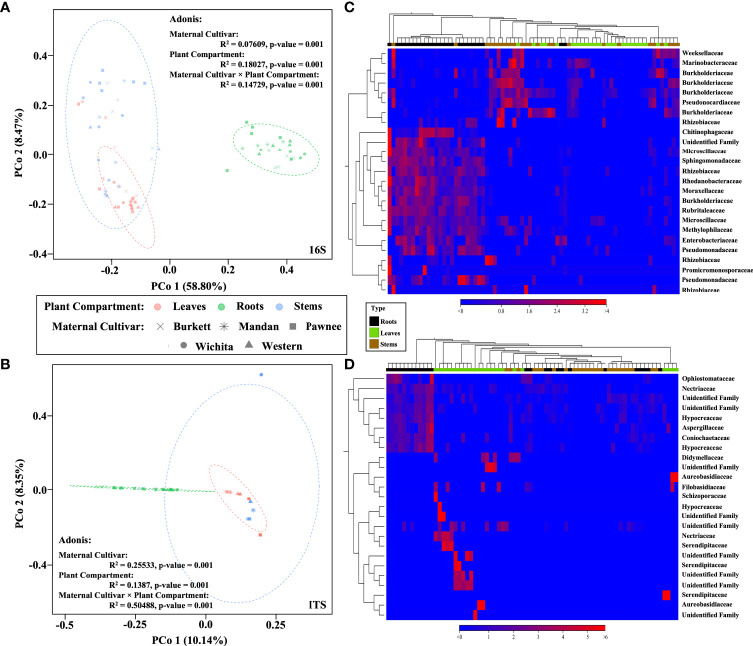
Bacterial and fungal community composition in pecan seedlings. PCoA visualization of bacteria **(A)** and fungi **(B)** communities present in pecan seedlings by maternal cultivar × plant compartment. The colored dots represent plant compartments (Leaf tissues are represented in peach, Stems in blue, and Roots in green) while the maternal cultivars are represented by different shapes (‘x’ is ‘Burkett’, * is ‘Mandan’, square is ‘Pawnee’, circle is ‘Wichita’, and triangle is ‘Western’). Heat map visualization of bacterial **(C)** and fungal **(D)** beta diversity of pecan seedlings compared by tissue type. Here we showed the 25 most abundant OTUs with their taxonomic assignment at the family level.

Beta diversity analysis revealed different clusters based on the similarities of tissue types for both bacterial and fungal communities (**Figure 2**). In order to better visualize the hierarchical clustering of the fungal communities present in pecan seedling tissues, a leaf sample (with insufficient reads) along with unknowns were removed. An Adonis test by tissue type revealed that the different clusters based on tissue types were statistically significant, not only in composition but in abundance as well. Bray-Curtis was used as measure for beta diversity when conducting Adonis tests of bacterial and fungal communities. These analyses lead to R^2^ values of 0.18027 (bacterial) and 0.1387 (fungal) with p-values of 0.001 for both bacterial and fungal communities (**Figures 2A, B**).

### Microbiome analysis by maternal cultivar indicate core microbiome

Sequence analysis of bacterial communities resulted in a total of 5,603 OTUs with the exclusion of mitochondria, chloroplast, unknowns, and ambiguous taxa. Of these OTUs, 1,800 were identified in seedlings derived from ‘Burkett’, 1,623 in ‘Mandan’ seedlings, 1,209 in ‘Pawnee’ seedlings, 1,443 in ‘Wichita’ seedlings, and 1,755 in ‘Western’ seedlings with a count of 241 OTUs shared across all seedlings. A total of 342 OTUs aggregated by family were observed; 234 were identified in ‘Burkett’ seedlings, 204 in ‘Mandan’ seedlings, 175 in ‘Pawnee’ seedlings, 206 in ‘Wichita’ seedlings, and 219 ‘Western’ seedlings. Analysis revealed 124 families shared across all seedlings (biological replicates) for all maternal cultivars. **Table 1** indicates the number of OTUs and families shared among the seedlings. Note that Table is arranged from the most 16S shared sequences to the least. Of the 428,370 fungal OTUs analyzed, 105,469 were identified in ‘Burkett’ seedlings, 60,359 in ‘Mandan’ seedlings, 83,557 in ‘Pawnee’ seedlings, 63,642 in ‘Wichita’ seedlings, and 115,558 in ‘Western’ seedlings with only 28 OTUs shared across all seedlings. A total of 94 OTUs aggregated by family were observed; 63 were identified in ‘Burkett’ seedlings, 54 in ‘Mandan’ seedlings, 50 in ‘Pawnee’ seedlings, 60 in ‘Wichita’ seedlings, and 51 in ‘Western’ seedlings. Analysis revealed 25 families were shared across all seedlings. Specific families shared between the seedling maternal cultivars are shown in **Table 1**. Furthermore, fungal analysis revealed that of the 428,370 OTUs, 402,803 were unknowns.

**Table 1:** OTUs and families shared between seedlings from different maternal cultivars.

Seedling Comparison	Shared 16S OTUs	Shared 16S Family	Shared ITS OTUs	Shared ITS Family
Burkett and Western	541	174	48	43
Burkett and Wichita	489	164	48	45
Wichita and Western	483	162	40	39
Mandan and Western	446	166	46	37
Burkett and Mandan	437	163	53	41
Mandan and Wichita	434	154	50	39
Pawnee and Western	406	143	37	35
Burkett and Pawnee	403	147	43	38
Pawnee and Wichita	397	142	41	37
Mandan and Pawnee	384	143	45	38

Number of OTUs and families shared among seedlings from different maternal cultivars for both bacterial and fungal communities present.

**Figure 3:**
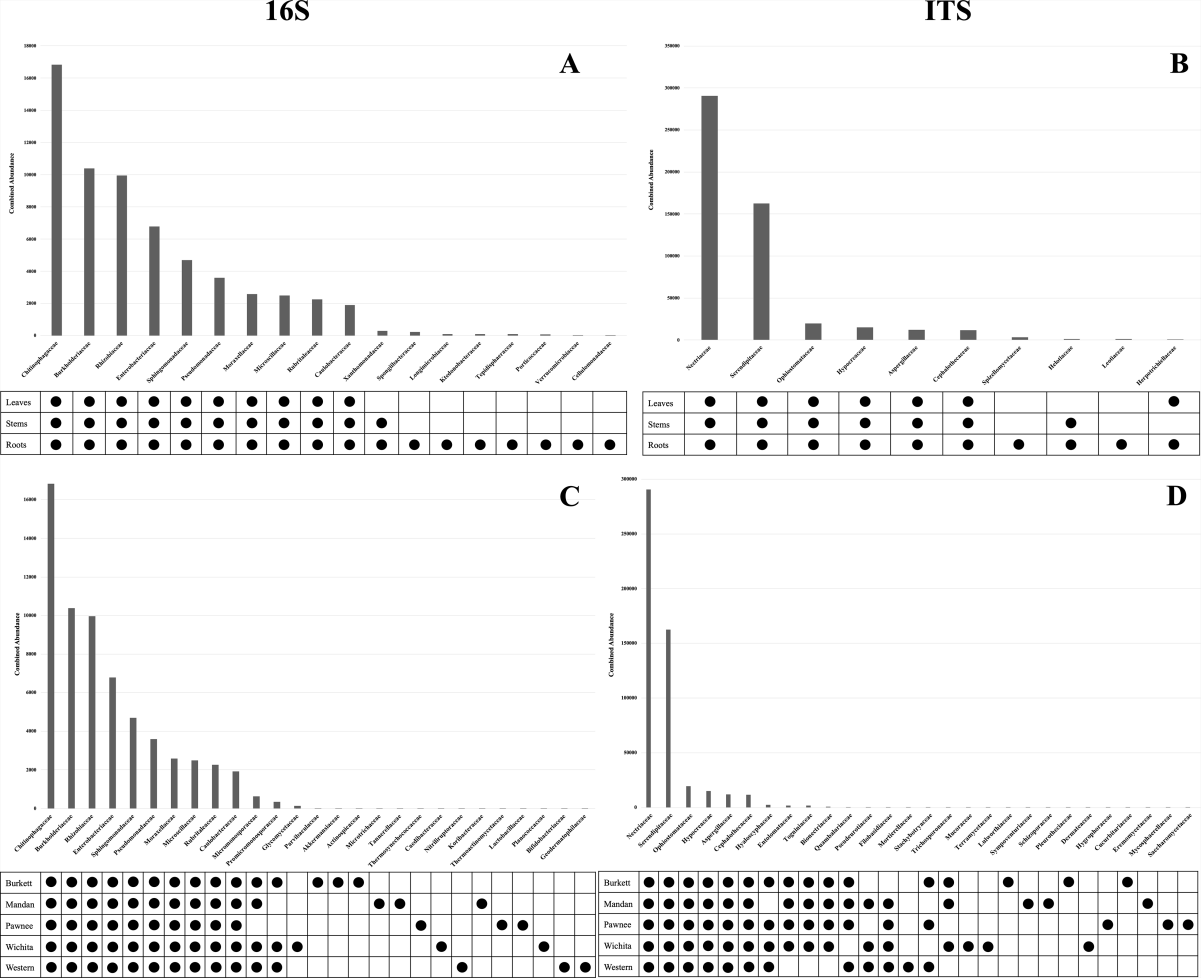
Core microbiome of pecan seedlings. **(A)** Bacterial and **(B)** fungal table showed the core microbial families in different seedling tissue types. **(C)** Bacterial and **(D)** fungal table showed the core microbial families in different maternal cultivars.

**Figure 4:**
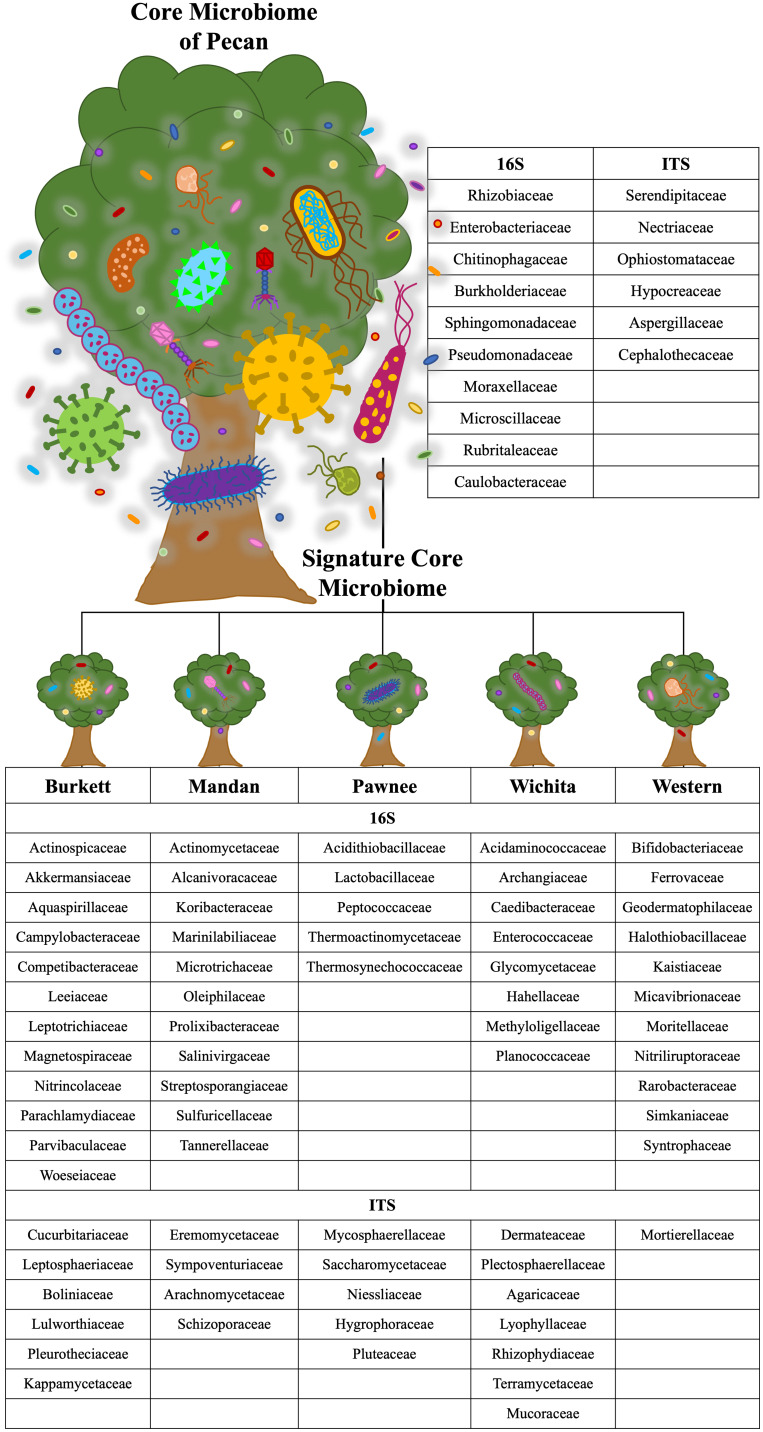
Core and cultivar specific signature microbiome. The core microbiome is shown with the proposed signature microbiome for each maternal pecan cultivar in this study. The bacterial and fungal families are listed.

Microbial population abundances and their presence between seedlings derived from pecan cultivars were revealed through bacterial and fungal sequence analysis and subsequent analyses (**Supplementary Figure S2**). Further analyses indicated a core microbiome, defined here as families found across all maternal cultivars, for pecan seedlings. Though most OTUs were unidentified at the genus level, the identifiable core genera from each member of the core families are listed in **Supplementary Table S2**. The following families were found to comprise the core microbiome, not only across maternal cultivars, but across seedling tissue type as well (**Figure 3**). Bacterial analysis revealed the families *Rhizobiaceae* (Pseudomonadota) and *Enterobacteriaceae* (Pseudomonadota) to be present in all seedlings with the ‘Pawnee’ seedlings having the most relative abundance. Conversely, the family *Chitinophagaceae* (Bacteroidota) was also found in all seedlings with the ‘Burkett’ seedlings having the most relative abundance followed by the seedlings from ‘Wichita’, ‘Western’, ‘Mandan’, and ‘Pawnee’. *Burkholderiaceae* (Pseudomonadota) was the most abundant in the ‘Mandan’ seedlings followed closely by ‘Western’, ‘Pawnee’, and ‘Wichita’ seedlings, with the ‘Burkett’ seedlings having the least relative abundance. Other top bacterial families identified across the pecan seedlings include *Sphingomonadaceae* (Pseudomonadota), *Pseudomonadaceae* (Pseudomonadota), *Moraxellaceae* (Pseudomonadota), *Microscillaceae* (Bacteroidota), *Rubritaleaceae* (Verrucomicrobia), and *Caulobacteraceae* (Pseudomonadota). Furthermore, specific bacterial families were identified to be respective to each maternal cultivar (**Figures 3C**, **4**). Within the core bacterial families identified, 52 core genera were identified to be shared across all maternal cultivar seedlings (**Supplementary Table S2**). Among these 52 identified genera, the top 10 include *Chitinophaga*, *Pseudomonas*, *Enterobacter*, *Luteolibacter*, *Sphingobium*, *Novosphingobium*, *Niastella*, *Caulobacter*, *Limnobacter*, and *Hylemonella*.

The family *Serendipitaceae* (Basidiomycota) was found to be most relatively abundant in the seedlings from the ‘Western’ maternal cultivar followed by seedlings from ‘Wichita’, ‘Pawnee’, ‘Burkett’, and ‘Mandan’. Interestingly, *Nectriaceae* (Ascomycota) was found to be most abundant in the ‘Burkett’ and ‘Western’ seedlings followed by ‘Mandan’, and ‘Pawnee’ with ‘Wichita’ seedlings having the least relative abundance. Other top fungal families identified across the seedlings include *Ophiostomataceae* (Ascomycota), *Hypocreaceae* (Ascomycota), *Aspergillaceae* (Ascomycota), and *Cephalothecaceae* (Ascomycota). As with bacteria, specific fungal families were identified to be respective to each maternal cultivar (**Figures 3D** and **4**). Within the core fungal families identified, only 10 core genera were identified to be shared across all maternal cultivar seedlings which include *Fusarium*, *Trichoderma*, *Penicillium*, *Neonectria*, *Aspergillus*, *Phialemonium*, *Meliniomyces*, *Cladophialophora*, *Corallomycetella*, *Xenocylindrocladium* (**Supplementary Table S2**). The OTUs from the core families were used for functional inference and the results for 16S and ITS can be found in **Supplementary Table S4**. The results using the EC database identified six functional enzymatic categories (oxidoreductases, transferases, hydrolases, lyases, isomerases, and ligases) for both bacterial and fungal families. The EC numbers divided into their sub-subclasses to the identified inferred enzyme can be found in **Supplementary Table S4**.

### Alpha diversity analysis by maternal cultivar

**Figure 5:**
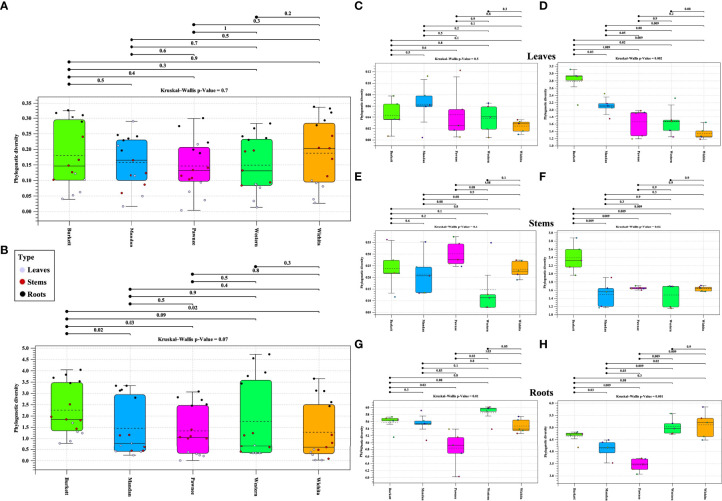
Phylogenetic alpha diversity of microbial populations in seedlings from five maternal pecan cultivars and between seedling tissue type. Box plot visualization of bacterial **(A)** and fungal **(B)** alpha diversity in pecan seedlings from different maternal cultivars. The colored dots indicate seedling tissue type samples per maternal cultivar. Leaf tissues are represented in purple, Stems in red, and Roots in black. Alpha diversity of tissue types: **(C)**16S and **(D)** ITS alpha diversity of leaves; **(E)** 16S and **(F)** ITS alpha diversity of stems; and **(G)** 16S and **(H)** ITS alpha diversity of roots.

Alpha diversity analysis of bacterial populations across seedlings derived from five maternal pecan cultivars in the study indicated ‘Burkett’ and ‘Wichita’ to have a similar range (min/max and interquartile) in diversity as well as ‘Mandan’ and ‘Western’ (**Figure 5A**). Furthermore, the seedlings within each maternal cultivar indicated a relationship of homogeneity among each other. Alpha diversity analysis of fungal populations revealed that seedlings from the ‘Western’ cultivar have a wider range in diversity compared to the other maternal pecan cultivar seedlings (**Figure 5B**). The seedlings from the ‘Burkett’, ‘Pawnee’, and ‘Wichita’ cultivars had a similar range in diversity with the seedlings from the ‘Pawnee’ cultivar having a tighter distribution. As observed with the bacterial communities, the seedlings in each maternal cultivar continued to follow a relationship of homogeneity. The p-values for both bacterial and fungal analysis indicated that there were no differences in alpha diversity between seedlings from different maternal cultivars (p-value = 0.7 and p-value = 0.07, respectively). Alpha diversity analyses comparing seedling tissue types (leaves, stems, roots) between maternal cultivars revealed differences (**Figures 5C–H**). Significant differences in the alpha diversity of bacterial communities were observed in the roots of seedlings within each respective cultivar while significant differences were observed in the fungal communities present across each tissue type.

### Beta diversity analysis by maternal cultivar

**Figure 6:**
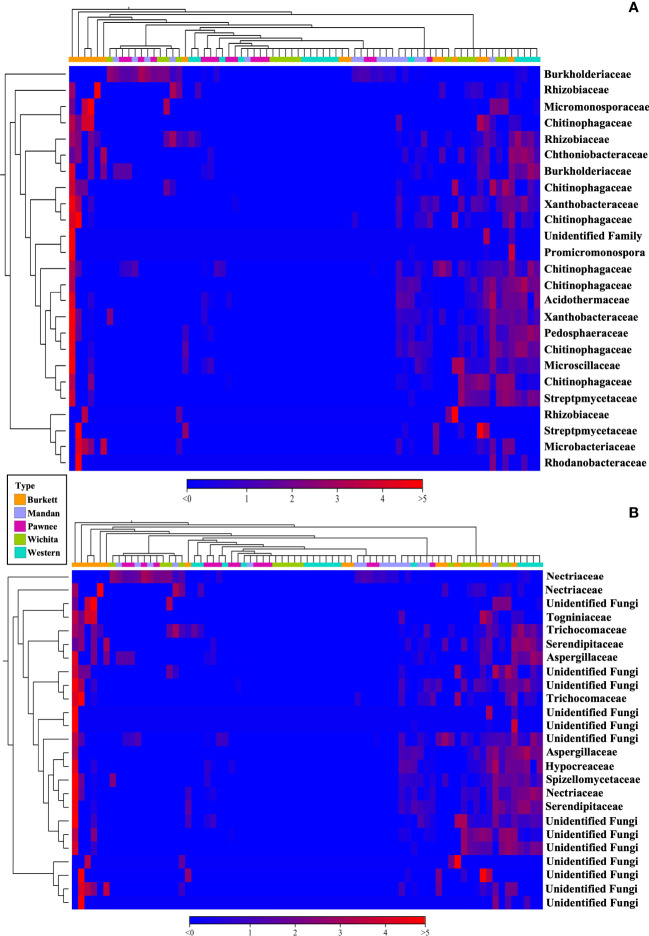
16S **(A)** and ITS **(B)** heat map visualization of beta diversity between seedlings from five maternal pecan cultivars. Here we showed the 25 most abundant OTUs with their taxonomic assignment at the family level.

Beta diversity analysis of bacterial communities revealed the seedlings to be dispersed with no apparent clustering based on the maternal cultivar (**Figure 2A**). On the other hand, beta diversity of fungal communities revealed seedlings from each the maternal cultivar to cluster more closely together with some dispersed seedling samples from the ‘Wichita’ cultivar tightly clustering together (**Figure 2B**). An Adonis test by maternal cultivar, using Bray-Curtis as measure, further revealed R^2^ values of 0.07609 (bacterial) and 0.25533 (fungal) with p-values of 0.001 for both bacterial and fungal communities (**Figures 2A, B**). Heat map analysis further demonstrated unique bacterial and fungal communities present in the seedling samples within their respective maternal cultivar (**Figure 6**). However, of the 25 most different fungal OTUs across all samples, only 2 families were identified as *Nectriaceae* and *Serendipitaceae*, 21 were unknowns, and 2 families were unidentified (**Supplementary Figure S3**). In order to observe differences between the identified fungal OTUs, unknowns were removed from the graphic (**Figure 6B**). Further Adonis tests measuring the effects that ‘maternal cultivar × plant compartment’ have on the microbial composition of pecan seedlings, revealed R^2^ values of 0.14729 (bacterial) and 0.50488 (fungal) with a p-value of 0.001 for both bacterial and fungal communities (**Figures 2A, B**). PERMANOVA analysis, using Bray-Curtis as a measure, revealed that the bacterial and fungal communities of seedlings within each respective maternal cultivar were not different to each other (p-value > 0.9). Differences in the beta diversity of bacterial communities were only observed in the roots of seedlings within each respective cultivar. In contrast, differences among the fungal communities across each tissue type were observed between seedlings within each maternal cultivar. Further pairwise comparisons testing the bacterial and fungal community compositions between the seedlings of different maternal cultivars revealed significant differences between some maternal cultivars (**Supplementary Table S3**).

## Discussion

### Microbial composition analysis of pecan seedlings

Bacterial and fungal OTUs differed in the presence/absence and relative abundance between pecan tissue types as previously reported on cottonwood trees (Cregger et al., 2018). These differences demonstrate that the pecan microbiome composition varies across tissue types (leaves, stems, roots). As these pecan seedlings were grown in a tightly regulated environment (i.e., planted in the same soilless potting mixture, grown in a quarantined greenhouse, and had the same water source) the differences between leaves, stems, and roots could be directly compared. Though the sources of seedling microbiome composition are yet to be further investigated, the possibility that bioaerosols and microorganisms present in water may contribute to plant microbiota should not be discarded. Microbial abundance and diversity were observed to be higher in the roots, as would be expected, possibly due to the direct interaction between roots and the potting mix. Limited Mi-Seq data and analyses of ‘Western’ pecan seeds (data not included) revealed a few families that were absent in the seeds when compared to the seedlings. Primer-specific PCR was performed on DNA extracted from Lamberts professional high porosity growing mix to determine if the few families that were missing in the seed (such as Rhizobiaceae and Chitinophagaceae) were present in the potting mix, and results indicated that these families were indeed in the potting mix. Even though the potting mix was treated with hydrogen peroxide, we surmise that the seedlings may have obtained these few families from the potting mix. Further studies analyzing the recruitment of microbes to pecan roots will be conducted. Roots not only take up mineral nutrients from the soils where they grow, but recruit microbes as well (Dastogeer et al., 2020). In addition to the type of soil and soil pH, carbon/nitrogen ratio along with available phosphorus and potassium in the soil, have been reported to influence the root microbial composition through their influence on plant growth and immunity (Chaparro et al., 2012; Bulgarelli et al., 2013; Turner et al., 2013). Thus, soil is a major factor contributing to root microbiome composition, as soils contain microbial communities themselves.

Of the bacterial families identified in pecan, *Moraxellaceae* was found to be more abundant in the leaves than in the stems and roots. This is consistent with previous studies on *Panax quinquefolius* and tomato (Romero et al., 2014; Shehata et al., 2021). *Moraxellaceae* belongs to the phyla Proteobacteria (commonly found in plant leaves), known to establish different interactions with plants such as mutualistic, parasitic, or neutral (Bulgarelli et al., 2013; Romero et al., 2014). Furthermore, *Chitinophagaceae* was found to be most abundant in the roots whereas *Rhizobiaceae* was most abundant in the stems. Studies on wild relatives of common bean also found *Chitinophagaceae* to be predominant in the root microbiome (Pérez-Jaramillo et al., 2018). Members of the *Chitinophagaceae* family can degrade organic matter such as chitin and cellulose (Rosenberg, 2014). In addition, research by Carríon et al. (2019) found that when sugar beet seedlings were inoculated with a root pathogen, the family *Chitinophagaceae*, along with *Flavobacteriaceae*, were enriched and correlated to disease suppression. Functional inference of sequences within these families found the possibility of disease suppression and nutrient acquisition but this would have to be verified in a further study (**Supplemental Table S4**). For instance, inferred functional analysis identified EC: 3.5.1.5 (**Supplementary Table S4**), in both bacterial and fungal core microbiomes, which corresponds to urease(s). Research on plant and bacterial ureases, have identified antifungal activities to phytopathogenic fungi (Becker-Ritt et al., 2007). Furthermore, ureolytic activity has been proposed to be involved in nitrogen bioavailability and protection against pathogens. EC number 3.5.2.6 was also identified in both bacterial and fungal core microbiomes corresponding to beta-lactamase (**Supplementary Table S4**). Research by Hossain et al. (2007), demonstrated the development of systematic resistance in *A. thaliana* to *Pseudomonas syringae* pv. tomato DC3000 through the interaction of *A. thaliana* with *Penicillium* secreted protease-sensitive molecules. Other studies evaluating the effects of beta-lactam antibiotics on plants reported the interaction between *A. thaliana* roots and soil fungi to be altered (Gudiño et al., 2018). The fungal family *Serendipitaceae* was found to be most abundant in the roots which, according to Weiß et al. (2016), interact diversely with plant roots. *Serendipitaceae* is a family known to serve as a beneficial growth-promoting fungus in a wide range of agricultural crops (Fritsche et al., 2021). This suggests that microbial presence and abundance in tissue types varies by plant species. As this study was performed with consistent conditions in a green house and same soil composition our results indicate a core microbial composition with the exclusion of several environmental factors.

### Comparison of microbial composition between seedlings derived from five different maternal cultivars reveals a core microbiome

In our study, overall bacterial and fungal microbiome differences were observed followed by comparisons in the presence and relative abundance of bacterial and fungal communities between seedlings from different pecan maternal cultivars. Under the same environmental conditions, microbial composition significantly varied between seedlings from each maternal pecan cultivar. While microbiome studies on pecan are new, our results are like those previously reported on the effects that plant genotypes, of the same species, have on microbiota assembly (Lundberg et al., 2012; Schlaeppi et al., 2014; Coleman-Derr et al., 2016). Although the seedlings in our study were grown using the same commercial potting mix in a regulated greenhouse, differences between seedlings from different maternal cultivars were still observed providing insight into the impact that genetic variation has on the core microbiome of pecans.

Our analyses on pecan seedlings identified a group of microbial communities that were, not only present in leaves, stems, and roots, but also shared across the seedlings from different Maternal cultivars (**Figure 3** and **Supplementary Table S2**). The bacterial families include *Rhizobiaceae*, *Enterobacteriaceae*, *Chitinophagaceae, Burkholderiaceae, Sphingomonadaceae, Pseudomonadaceae, Moraxellaceae, Microscillaceae, Rubritaleaceae, Caulobacteraceae*; and fungal families include *Serendipitaceae, Nectriaceae, Ophiostomataceae, Hypocreaceae, Aspergillaceae, and Cephalothecaceae.* Thus, providing insight into the core microbiome of pecan. As we further analyzed the microbiome of pecan seedlings from five maternal cultivars, we found significant differences between them indicating distinct cultivar-specific microbiome signatures (**Figure 4**). Had the study been continued it is likely that microbial changes may have occurred as the seedlings continued to grow into maturity as observed in Marques et al. (2014), where bacterial composition in three sweet potato cultivars changed as the plants developed and aged. These findings were attributed to root growth, architecture, morphology, physiology, composition, and the production of specific phytochemicals produced by root exudates known to be distinct at different plant developmental stages influencing microbial communities in plants (Chaparro et al., 2014; Sugiyama et al., 2014; Pérez-Jaramillo et al., 2017).

Though our findings were in seedlings, nonetheless, they are similar to those of Adam et al. (2018), where genotype-specific microbiomes were identified in the seeds of *Cucurbita pepo* (pumpkin). Other recent similar reports on genotype specific microbiomes have also been published on the seeds of *Lens culinaris* (lentils) (Morales Moreira et al., 2021). While the main contributor to the microbiome of seeds is not fully understood, the vertical transmission of microorganisms from plant parents to seeds has been previously reported by Berg and Raaijmakers (2018). Zaumeyer (1930) traced the passage of *Xanthomonas axonopodis pv. phaseoli* from bean plants to pods and found that the bacteria in the xylem vessels, of the stem, travels through the vascular system by way of the raphe and into the seed coat. Once in the seed coat, *Xanthomonas* travels through the intercellular spaces and spreads throughout the tissue of the seed (Zaumeyer, 1930). Seed to seedling transmission of microorganisms has also been reported in pecans with *Xylella fastidiosa* (Cervantes et al., 2022). A study by Brinkerhoff and Hunter (1963), found *Xanthomonas malvacearum* to be present in the embryos and seed coats of cotton. *Xanthomonas campestris* was also found to be efficiently transmitted from seed to seedlings from inoculated bean flowers (Darrasse et al., 2010). In the previously mentioned limited data on ‘Western’ seeds, not included here, nut shells were removed, and DNA was extracted only from the nut kernels. Thus, studies investigating the microbiome of pecan seeds is to be further explored.

In the field study by Walters et al. (2018), heritable microbes were identified in the rhizosphere of different maize genotypes in which certain taxa were host genotype specific. Plant genetic effects on the rhizosphere microbiome were found to be significant regardless of the plant’s age, specific communities present in the fields, and the response that microbial communities had on climatic events (Walters et al., 2018). In our study, significant differences in microbial abundances between seedlings from different maternal cultivars were irrefutable. For example, 12 bacterial families were identified to be unique to the seedlings from the ‘Burkett’ cultivar compared to those of the ‘Pawnee’ cultivar with 5 unique families identified in the seedlings, etc. (**Figure 4**). Furthermore, fungal families unique to each maternal cultivar were also identified (**Figure 4**). Bacterial families *Rhizobiaceae* and *Enterobacteriaceae* were identified in all seedlings with the seedlings from the ‘Pawnee’ cultivar having the most relative abundance, while *Chitinophagaceae* was identified to be most relatively abundant in the seedlings from the ‘Burkett’ cultivar. Indicating the influence that maternal cultivars have on the resulting progeny.

Research by Cregger et al. (2018) looked at the microbial communities present in the leaves, stems, and roots of *Populus*; and found roots to have the highest archaeal and bacterial diversity compared to fungal diversity which was found to be highest in the stems. In our study, significant differences in microbial diversity between leaves, stems, and roots; and seedlings from different maternal cultivars were observed. However, while the stronger factor influencing bacterial community composition in pecan seedlings was plant compartment, fungal communities were revealed to be influenced more by maternal cultivar (**Figures 2A, B**). This previous study further supports our supposition that different pecan cultivars hold their own signature microbiome regardless of the plant tissue type. Furthermore, it is important to note that no differences between the seedlings within each respective maternal cultivar were observed, suggesting a cultivar-specific signature core microbiome. These data infer a higher level of influence that maternal plants have on the microbiome composition of seeds and the resulting seedlings as the core microbiome may be inheritable through genetics and vertical transmission (Liu et al., 2017; Bhagat et al., 2021), as well as the plant microbiome composition being modulated by seeds (Moroenyane et al., 2021).

The observed microbiome differences between pecan genotypes may account, to a certain degree, for the unique characteristics that each pecan cultivar has such as nutrient acquisition, resistance/susceptibility to pathogenic microorganisms, and environmental stressors (i.e., drought). Further studies would need to be performed to correlate specific microbiome members to specific phenotypes. For dry areas with limited water access, such as the Southwest US, drought tolerance would be a characteristic of high value for pecan growers. Research by Marasco et al. (2013), revealed an increase in drought tolerance and an enhanced root system, that improved water uptake, in pepper plants inoculated with a desert origin bacterium. In our study, among the 25 most different OTUs across all samples, *Serendipitaceae* was identified in the ‘Mandan’ seedling samples (**Supplementary Figure S3**). Though the genus *Serendipita* was not part of the pecan seedling core genera, research by Ray et al. (2021) found *Serendipita* fungi to bypass the defense mechanisms of switchgrass by modulating the root transcriptome and establishing a symbiotic relationship. *Serendipitaceae* species are known for the beneficial effects they have on host plants such as enhanced resistance to abiotic and biotic stressors (Weiß et al., 2016). In addition, *Serendipitaceae* are widely known to serve as growth-promoting fungi for a broad range of agricultural crops (Jacquemyn et al., 2015; Reiter et al., 2020; Fritsche et al., 2021). Nonetheless, the results of our study should facilitate expanded studies on the establishment and species identification of the microorganisms that form the pecan core microbiome. This in turn, will provide additional information to pecan breeders that seek to create a pecan genotype with the appropriate characteristics and microbiomes needed to thrive under specific environmental conditions. Further research looking at the maternal, seed, and seedling microbiome at different developmental stages into maturity should be conducted to further our understanding of the pecan microbiome. By elucidating the heritable plant-microbe interactions in pecan, such interactions may ultimately be incorporated into the breeding practices for pecan.

## Data availability statement

The datasets presented in this study can be found in online repositories. The names of the repository and accession numbers can be found below: National Center for Biotechnology Information (NCBI) BioProject, https://www.ncbi.nlm.nih.gov/bioproject/, PRJNA796047 and PRJNA796064.

## Author contributions

KC, RH, and JR conceived and designed the study. KC performed the experiments. JR and RH contributed working space, materials, and equipment. JR provided funding for the research. KC and JR wrote the manuscript. All authors contributed to the article and approved the submitted version.

## Funding

Funding from USDA-NIFA-SCRI 2016-51181-25408.

## Acknowledgments

The authors would like to acknowledge Liam St. Hilaire, Paul Lambert, Esteban Molina, and Paul Gabriel for their help in DNA extraction. The authors would like to acknowledge and thank Dr. Ciro Velazco-Cruz for his help with statistical analysis. The authors would also like to acknowledge the NMSU Experimental Station and the NMSU experimental farm Leyendecker for their support.

## Conflict of interest

The authors declare that the research was conducted in the absence of any commercial or financial relationships that could be construed as a potential conflict of interest.

## Publisher’s note

All claims expressed in this article are solely those of the authors and do not necessarily represent those of their affiliated organizations, or those of the publisher, the editors and the reviewers. Any product that may be evaluated in this article, or claim that may be made by its manufacturer, is not guaranteed or endorsed by the publisher.

## Supplementary material

Supplementary Table 1 Pecan cultivars and their respective characteristics. Data obtained from Thompson & Young, 1985; Sparks, 1992.

Supplementary Table 2 Excel sheet with multiple tabs providing information on the bacterial and fungal core genera identified within the core microbial families that comprise the pecan core microbiome.

Supplementary Table 3 PERMANOVA analysis comparing the microbial communities between seedlings from one maternal cultivar to those of another maternal cultivar.

Supplementary Table 4 Excel sheet with multiple tabs providing information on the inferred functions of bacterial and fungal families that comprise the pecan core microbiome.

Supplementary Figure 1 Relative abundance of microbial populations between pecan tissues. Stacked bar chart visualization of OTU clustering at the family level of bacterial **(A)** and fungal **(B)** microbial communities in pecan.

Supplementary Figure 2 Relative abundance of microbial populations between seedlings from five maternal pecan cultivars. Stacked bar chart visualization of OTU clustering at the family level of bacterial **(A)** and fungal **(B)** microbial communities in pecan.

Supplementary Figure 3 ITS beta diversity – heat map of fungal communities in seedlings from five maternal pecan cultivars. Heat map visualizes fungal beta diversity of pecan seedlings with unknowns; supplementary to **Figure 5B**.
