# Supplementary material for: The core microbiome of Carya illinoinensis (pecan) seedlings of different maternal pecan cultivars from the same orchard
Source: Front Microbiomes. 2022 Nov 11;1:1003112. doi: 10.3389/frmbi.2022.1003112 (PMC12993460; doi:10.3389/frmbi.2022.1003112)
Supplement: Supplementary Table 1 — Pecan cultivars and their respective characteristics. Data obtained from Thompson & Young, 1985; Sparks, 1992. [file DataSheet_1.zip › Supplementary Table 1.pdf]

	Susceptibility and Resistance	Planting Recommendation	Nut	Kernel
**Burkett**	• Very susceptible to scab<br>• Very susceptible to downy spot	• Widely used as nursery seedstock for the western pecan growing region	• Orbicular, with obtuse apex and rounded base<br>• Round in cross section	Golden to light brown in color with prominent dark brown speckles.
**Mandan**	• Resistant to scab disease<br>• Medium susceptibility to yellow and black aphids	• N/A but should be a good pollinizer for, and be well pollinized by 'Kanza', 'Wichita', and 'Lakota'	• Oblong elliptic with an obtuse apex<br>• Rounded base and is flattened in cross section	Cream to golden in color, with medium, non-trapping dorsal grooves and rounded dorsal ridge.
**Pawnee**	• Medium susceptibility to scab<br>• Fair resistance to downy spot<br>• Resistant to yellow aphids	• TX, OK, KS, AL, AR	• Elliptical with obtuse apex and rounded base<br>• Laterally compressed in cross section	Golden in color with wide dorsal grooves and deep basal cleft.
**Western**	• Very susceptible to scab<br>• Very susceptible to downy spot	• AZ, CA, NM, OK, west TX	• Oblong elliptic to oblong, with right angled apex and acute base<br>• Asymmetric<br>• Round in cross section<br>• Rough shell surface	Golden to light brown, with deep, tight dorsal grooves that trap packing material and cause kernels to break during shelling.
**Wichita**	• Very susceptible to scab	• AZ, AR, CA, NM, OK, TX	• Oblong, with acute to acuminate, asymmetric apex and rounded apiculate base<br>• Round in cross section	Golden to light brown in color with narrow dorsal grooves and a wide, shallow basal cleft.
