# Supplementary material for: The core microbiome of Carya illinoinensis (pecan) seedlings of different maternal pecan cultivars from the same orchard
Source: Front Microbiomes. 2022 Nov 11;1:1003112. doi: 10.3389/frmbi.2022.1003112 (PMC12993460; doi:10.3389/frmbi.2022.1003112)
Supplement: Supplementary Table 1 — Pecan cultivars and their respective characteristics. Data obtained from Thompson & Young, 1985; Sparks, 1992. [file DataSheet_1.zip › Supplementary Table 3.pdf]

PERMANOVA – Bray Curtis					
**16S**	‘Burkett’	‘Mandan’	‘Pawnee’	‘Wichita’	‘Western’
‘Burkett’	-	-	-	-	-
‘Mandan’	0.03954	-	-	-	-
‘Pawnee’	0.01218	0.15304	-	-	-
‘Wichita’	0.30308	0.33411	0.05290	-	-
‘Western’	0.04453	0.06533	0.02006	0.06798	-
**ITS**	‘Burkett’	‘Mandan’	‘Pawnee’	‘Wichita’	‘Western’
‘Burkett’	-	-	-	-	-
‘Mandan’	0.00001	-	-	-	-
‘Pawnee’	0.00001	0.00001	-	-	-
‘Wichita’	0.00001	0.00001	0.00001	-	-
‘Western’	0.00001	0.00001	0.00001	0.00001	-
