# Supplementary figures and images for: The core microbiome of Carya illinoinensis (pecan) seedlings of different maternal pecan cultivars from the same orchard
Source: Front Microbiomes. 2022 Nov 11;1:1003112. doi: 10.3389/frmbi.2022.1003112 (PMC12993460; doi:10.3389/frmbi.2022.1003112)

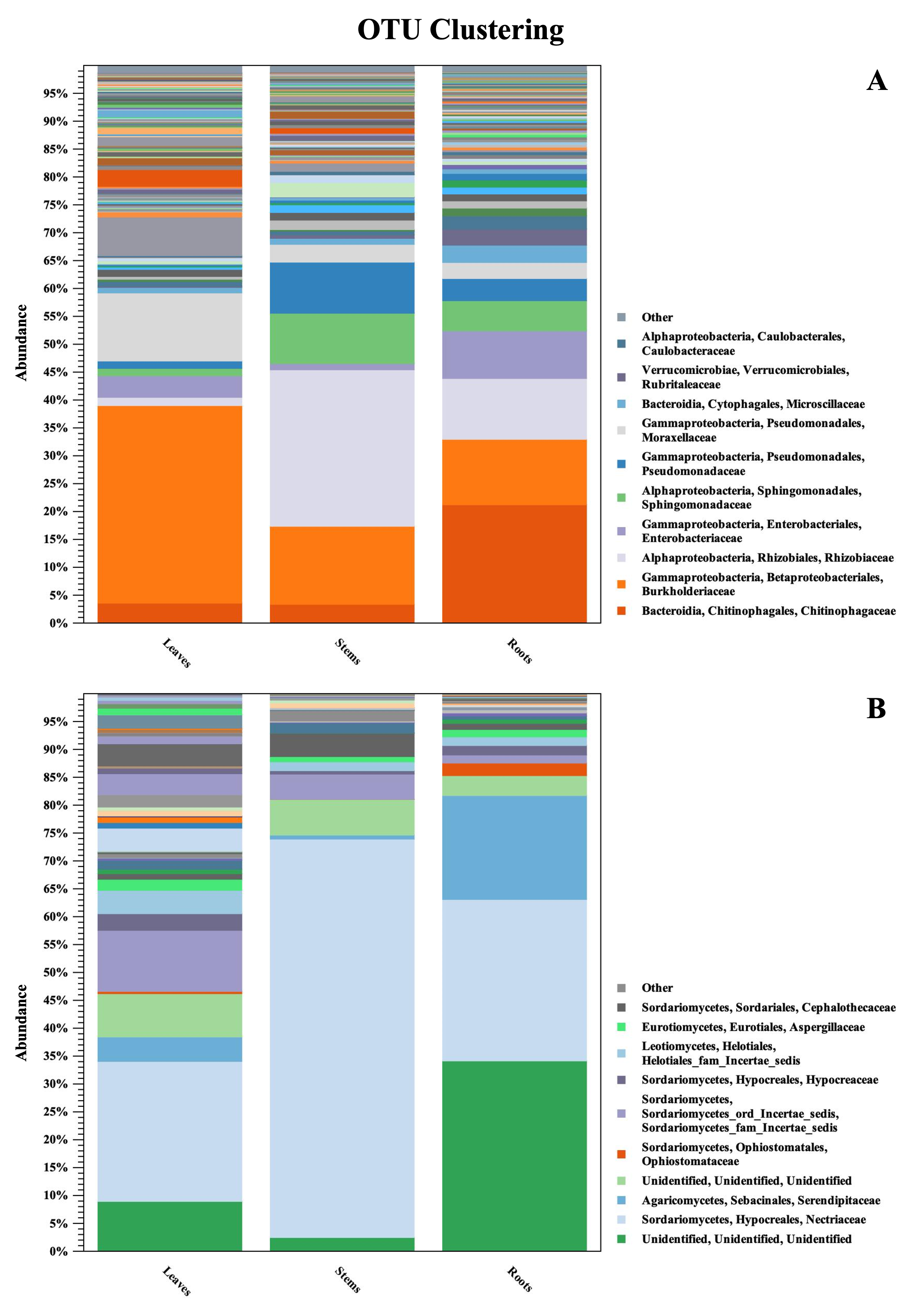

Supplement: Supplementary Table 1 — Pecan cultivars and their respective characteristics. Data obtained from Thompson & Young, 1985; Sparks, 1992. [file DataSheet_1.zip › Supplementary Figure 1.JPEG]

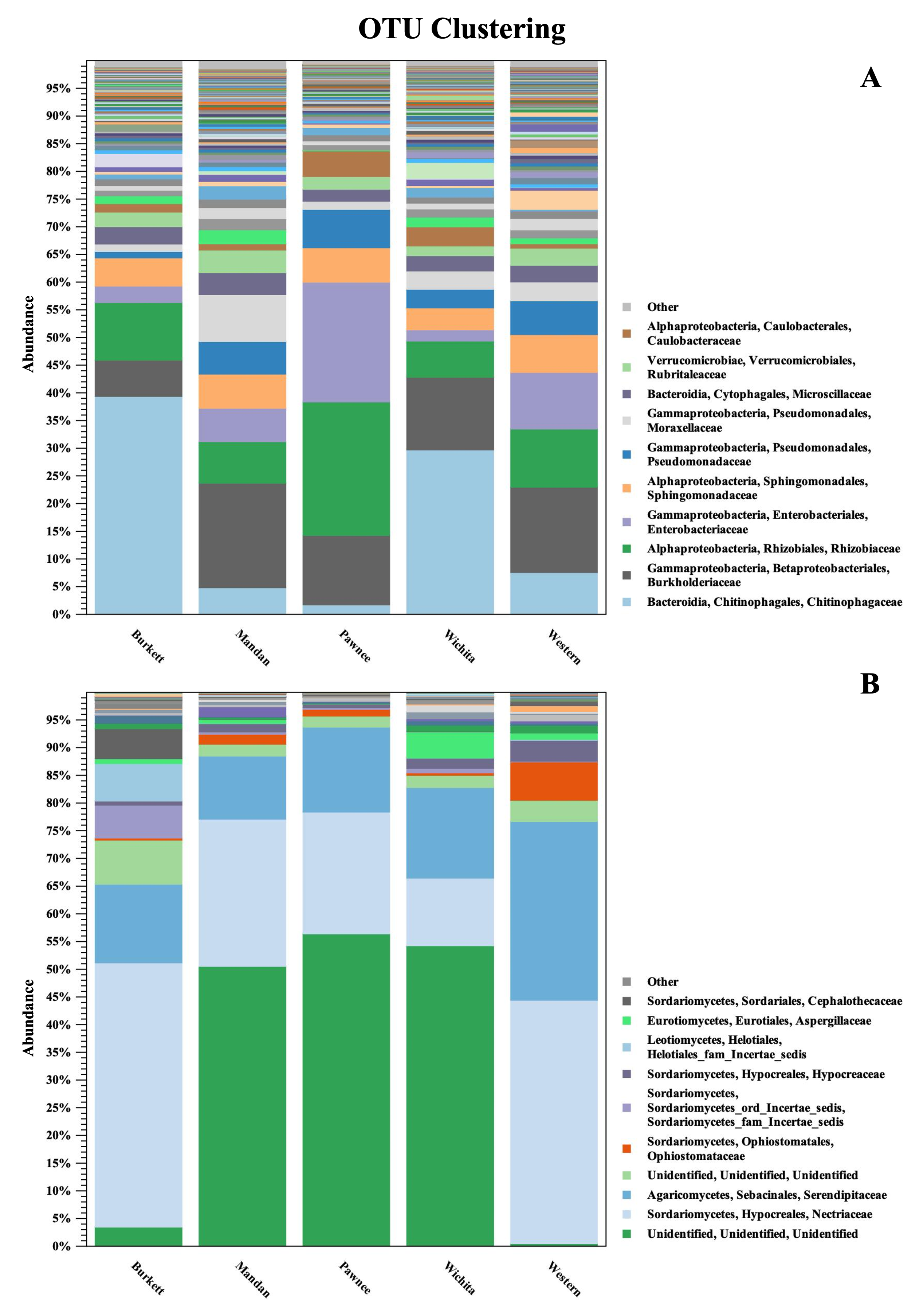

Supplement: Supplementary Table 1 — Pecan cultivars and their respective characteristics. Data obtained from Thompson & Young, 1985; Sparks, 1992. [file DataSheet_1.zip › Supplementary Figure 2.JPEG]

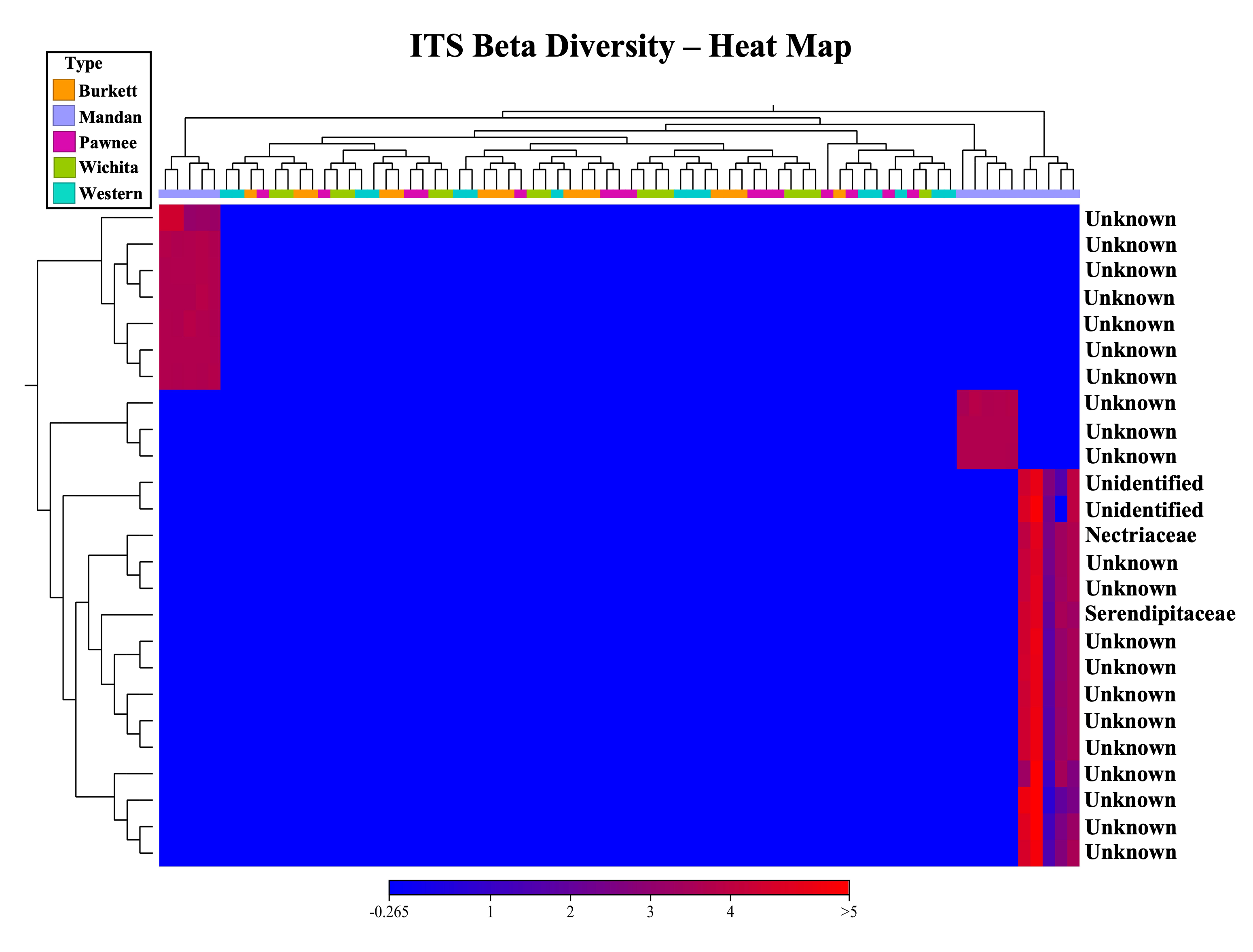

Supplement: Supplementary Table 1 — Pecan cultivars and their respective characteristics. Data obtained from Thompson & Young, 1985; Sparks, 1992. [file DataSheet_1.zip › Supplementary Figure 3.JPEG]
